# Pilot study to define criteria for Pituitary Tumors Centers of Excellence (PTCOE): results of an audit of leading international centers

**DOI:** 10.1007/s11102-023-01345-0

**Published:** 2023-08-28

**Authors:** A. Giustina, M. M. Uygur, S. Frara, A. Barkan, N. R. Biermasz, P. Chanson, P. Freda, M. Gadelha, U. B. Kaiser, S. Lamberts, E. Laws, L. B. Nachtigall, V. Popovic, M. Reincke, C. Strasburger, A. J. van der Lely, J. A. H. Wass, S. Melmed, F. F. Casanueva

**Affiliations:** 1 Institute of Endocrine and Metabolic Sciences, San Raffaele Vita-Salute University and IRCCS Hospital, Via Olgettina 60, 20132 Milan, Italy; 2 Department of Endocrinology and Metabolism Disease, School of Medicine, Recep Tayyip Erdoğan University, Rize, Turkey; 3 Division of Endocrinology, University of Michigan Health System, Ann Arbor, MI USA; 4 Leiden University Medical Center, Center for Endocrine Tumors Leiden, Leiden, The Netherlands; 5 Université Paris-Saclay, Inserm, Physiologie et Physiopathologie Endocriniennes, APHP, Hôpital Bicêtre, Service d’Endocrinologie et des Maladies de la Reproduction et Centre de Référence des Maladies Rares de l’Hypophyse HYPO, Le Kremlin-Bicêtre, Paris, France; 6 Department of Medicine, Vagelos College of Physicians and Surgeons, Columbia University, New York, NY USA; 7 Instituto Estadual do Cérebro Paulo Niemeyer, Secretaria Estadual de Saúde do Rio de Janeiro, Rio de Janeiro, Brazil; 8 Brigham and Women’s Hospital, Harvard Medical School, Boston, MA USA; 9 Erasmus Medical Center, Rotterdam, The Netherlands; 10 Pituitary/Neuroendocrine Center, Brigham & Women’s Hospital, Boston, MA USA; 11 Neuroendocrine Unit, Massachusetts General Hospital, Harvard Medical School, Boston, MA USA; 12 Medical Faculty, University of Belgrade, Belgrade, Serbia; 13 Department of Medicine IV, LMU University Hospital, LMU Munich, Munich, Germany; 14 Department of Medicine for Endocrinology, Diabetes and Nutritional Medicine, Charité Universitätsmedizin, Berlin, Germany; 15 Pituitary Center Rotterdam, Endocrinology Section, Department of Internal Medicine, Erasmus University Medical Center, Rotterdam, The Netherlands; 16 Department of Endocrinology, Churchill Hospital, University of Oxford, Oxford, United Kingdom; 17 Pituitary Center, Department of Medicine, Cedars-Sinai Medical Center, Los Angeles, CA USA; 18 Division of Endocrinology, Santiago de Compostela University and Ciber OBN, Santiago, Spain

**Keywords:** Acromegaly, Cushing’s disease, Prolactinoma, Centers of Excellence, Pituitary tumors, Criteria, Neurosurgery, Medical treatment

## Abstract

**Purpose:**

The Pituitary Society established the concept and mostly qualitative parameters for defining uniform criteria for Pituitary Tumor Centers of Excellence (PTCOEs) based on expert consensus.

Aim of the study was to validate those previously proposed criteria through collection and evaluation of self-reported activity of several internationally-recognized tertiary pituitary centers, thereby transforming the qualitative 2017 definition into a validated quantitative one, which could serve as the basis for future objective PTCOE accreditation.

**Methods:**

An ad hoc prepared database was distributed to nine Pituitary Centers chosen by the Project Scientific Committee and comprising Centers of worldwide repute, which agreed to provide activity information derived from registries related to the years 2018–2020 and completing the database within 60 days. The database, provided by each center and composed of Excel® spreadsheets with requested specific information on leading and supporting teams, was reviewed by two blinded referees and all 9 candidate centers satisfied the overall PTCOE definition, according to referees’ evaluations. To obtain objective numerical criteria, median values for each activity/parameter were considered as the preferred PTCOE definition target, whereas the low limit of the range was selected as the acceptable target for each respective parameter.

**Results:**

Three dedicated pituitary neurosurgeons are preferred, whereas one dedicated surgeon is acceptable. Moreover, 100 surgical procedures per center per year are preferred, while the results indicated that 50 surgeries per year are acceptable. Acute post-surgery complications, including mortality and readmission rates, should preferably be negligible or nonexistent, but acceptable criterion is a rate lower than 10% of patients with complications requiring readmission within 30 days after surgery. Four endocrinologists devoted to pituitary diseases are requested in a PTCOE and the total population of patients followed in a PTCOE should not be less than 850. It appears acceptable that at least one dedicated/expert in pituitary diseases is present in neuroradiology, pathology, and ophthalmology groups, whereas at least two expert radiation oncologists are needed.

**Conclusion:**

This is, to our knowledge, the first study to survey and evaluate the activity of a relevant number of high-volume centers in the pituitary field. This effort, internally validated by ad hoc reviewers, allowed for transformation of previously formulated theoretical criteria for the definition of a PTCOE to precise numerical definitions based on real-life evidence. The application of a derived synopsis of criteria could be used by independent bodies for accreditation of pituitary centers as PTCOEs.

**Supplementary Information:**

The online version contains supplementary material available at 10.1007/s11102-023-01345-0.

## Introduction

Symptomatic pituitary adenomas are relatively rare and largely underdiagnosed conditions, which may have serious systemic consequences [1]. Therefore, their diagnosis and management still represent a significant challenge for clinicians. Affected patients often suffer from severe and chronic systemic complications, poor quality of life and reduced life expectancy [2–4].

The “Centers of Excellence” concept is common to conditions that have low prevalence or require complex interventions, and of paramount interest not only to health professionals and patients, but also to health administrators. In fact, it is thought that the optimal and even the most cost-effective care for patients may be provided only by dedicated expert teams. Patients with rare conditions frequently have difficulties in finding a referral center for their disease and they and their families may have concerns about the quality of care that can be provided. This concept is particularly important in clinical activities needing multidisciplinary teams, which require both expert surgeons and specialized medical personnel [5–7].

A Pituitary Society-driven consensus has been developed based on the concept that care for patients bearing pituitary adenomas should be organized by Centers of Excellence, where neurosurgeons experienced in pituitary surgery and endocrinologists devoted to pituitary medicine are working closely with colleagues with specific experience in supporting units [8]. However, while several hospitals have already developed teams providing effective high-level interdisciplinary care, which theoretically represent the basis for a Pituitary Tumors Center of Excellence (PTCOE), most of these groups are self-appointed and without formal recognition by local or national health authorities. Moreover, a formal, internationally accepted definition of excellence is lacking for pituitary medicine with consequent difficulties in benchmarking activity of existing or yet-to-be formed centers due to lack of objective measures of patients’ outcomes. Importantly, once objective criteria are defined, it may be possible for external independent certifying organizations, recognized by health authorities worldwide, to provide formal accreditation of PTCOEs based on such criteria. This will avoid self-appointment or self-designation by unqualified centers without objective evaluation of their activity, external certification and assurance of quality of care provided. This process will fulfill the ultimate goal of conveying standardized, quality-controlled information to pituitary patients.

Previously, the Pituitary Society established the concept and primarily qualitative parameters for defining PTCOEs, in an attempt to provide uniform criteria for excellence of centers dealing with pituitary disorders [9–11]. Although those proposed criteria were based on expert consensus, due to their mainly theoretical nature, they still required to be tested in clinical practice prior to their possible widespread application.

The goal of this pilot study was to validate previously proposed criteria through collection and evaluation of self-reported activity of several internationally recognized tertiary pituitary centers. Moreover, the study aimed at providing a step towards formal PTCOE definition, transforming the qualitative 2017 approach into a quantitative one, which could serve as the basis for future objective PTCOE accreditation.

## Materials and methods

The pilot project for validation of criteria for accreditation of PTCOE followed a multi-step process which was expected to start in December 2019, but due to the coronavirus disease 2019 (COVID-19) [12] it was rescheduled to start on January 2021. The various pre-planned phases of this project are schematically summarized in Table 1.

**Table 1:** Pre-planned phases of the PTCOE criteria validation project

Pre-test phase (SEB constitution, database creation, centers selection
↓ Phase 1 (data submission from candidate centers) ↓ Phase 2 (internal data review) ↓ Phase 3 (re-submission of revised data from candidate centers) ↓ Phase 4 (review process) ↓ Phase 5 (collection of referee evaluations and data analysis) ↓
Phase 6 (sharing of data with the SEB and Centers and writing of final report)

In the pre-test phase Principal Investigator (PI) and Co-PIs agreed with the Pituitary Society on 12 experts (endocrinologists and neurosurgeons) designated to be balanced by geography and gender to serve as the Scientific Evaluating Board (SEB) [9].

The subsequent step was to design a database template according to criteria for PTCOE definition published in 2017 by The Pituitary Society Expert Group [9], which reflected the PTCOE mission of providing optimal care through a multidisciplinary team, led by neurosurgeons and endocrinologists in close collaboration with supporting units. The resulting database reviewed and approved by the SEB, consisted of several Excel® spreadsheets covering general information on the center and specific questions on Neurosurgery, Endocrinology, Neuroradiology, (Neuro-) Pathology and Radiotherapy Units, as well as other supporting units (Supplemental Table 1).

In the subsequent phase, the SEB was asked to anonymously identify candidate centers for the study, recognized for their expertise and, therefore, likely being putative PTCOEs. Those Centers receiving the most votes were contacted by the SEB: 9 of 10 agreed to provide activity information for 2018–2020 and committed to completing the database within 60 days. Four out of nine centers were represented on the SEB.

S.F. and M.M.U. oversaw an internal review upon receipt of all databases submitted by the centers before data analysis. Eight of the nine contained errors, missing or conflicting data and centers were asked to amend their files and/or provide missing data. Moreover, centers were asked to give feedback about the size of the database as well as the feasibility and challenges in collecting requested data.

In the next phase, two SEB members blinded to Centers were randomly assigned to review data provided by each Center. Referees were asked to provide their comments and complete an evaluation form (Supplemental Table 2) using a ranking system from 1 (lowest) to 5 (highest). A ranking ≥ 3 was considered positive for each item evaluated, including center facilities and clinical and educational multidisciplinary activities. Referees also evaluated each unit, giving scores for clinical, research, dissemination activities and trials of Endocrinology and Neurosurgery Units, the latter being evaluated also on post-surgical complications. Moreover, facilities of the neuroradiology and neuropathology units, as well as clinical activities of neuroradiology and radiotherapy units were evaluated by the referees. Upon completion of their review, the referees evaluated if the overall definition of PTCOE was satisfied, producing general (open) comments on the centers. Importantly, all 9 of 9 candidate centers satisfied the overall definition of PTCOE, according to referees.

In last phase of this pilot study after collecting all referee responses (3 were not received and were completed instead by M.M.U.), data analysis was completed by the end of July 2022. Core results were subsequently discussed with all SEB members and participating centers during a web call. During this call, SEB members highlighted some discrepancies/inconsistencies in the results. Accordingly, specific requests for revision were sent to participating centers, who all replied within 15 days. A representative of each center not already included in the SEB was invited to join the manuscript writing committee.

The results reported below include all final data from the nine candidate centers and are presented as total and percentage or as median and range. To avoid possible selection bias, also related to pandemic disruptions which occurred in many countries in 2020, we asked for data from years 2018, 2019 and 2020 which were later analyzed together (unless otherwise stated).

To obtain objective, numerical criteria, median values for each considered activity/parameter were considered as the preferred target in a PTCOE evaluation, whereas the low limit of the range for each parameter was selected as the acceptable target for each specific activity.

## Results

### Timing of center response and referee evaluation

Median time for data submission from candidate centers was 58 (37–89) days. After the internal review process, a revised database was submitted within a median time of 11 (4–81) days. The referee evaluation phase lasted 12 (3–65) days. A maximum score (equal to 5) for general and specific activity data was obtained for each center. Among the feedback received from candidate centers about the process, the most common comments were related to the very large dimension of the database and the difficulty in collecting all required information.

### Core activity data included in the PTCOE criteria

Core activity data collected for neurosurgical, endocrine and support units are reported below.

#### Neurosurgery

##### Personnel and volume of activity

**Fig. 1:**
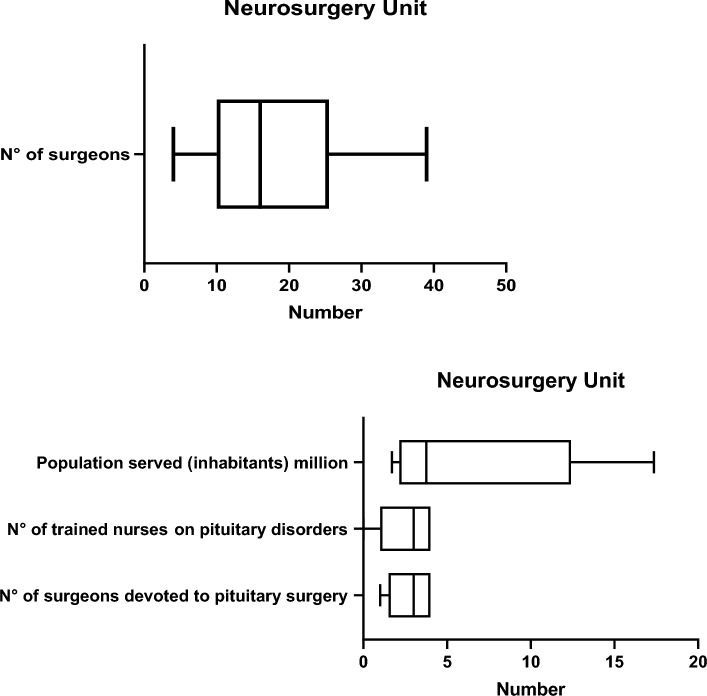
Personnel and population served in Neurosurgery Units

The median (min–max) number of surgeons in the Neurosurgery Department was 16 (4–39) and median number of neurosurgeons dedicated to pituitary surgery was 3 (1–4). There were 3 (0–4) trained pituitary nurses in the neurosurgery unit. The median population served was 3.76 (1.7–17.3) millions (Fig. 1).

**Fig. 2:**
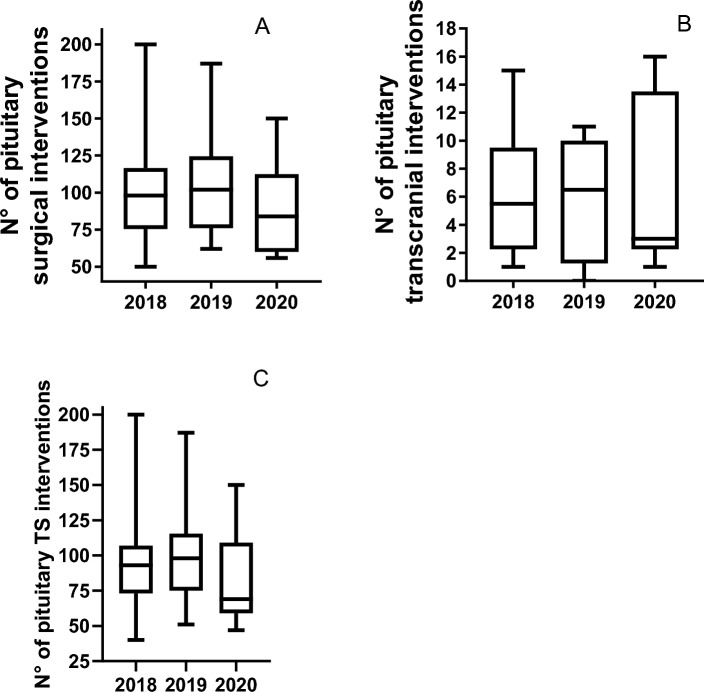
Pituitary Interventions per center per year (**A**) and per type of intervention [**B** transcranial and **C** transsphenoidal (TS)]

**Fig. 3:**
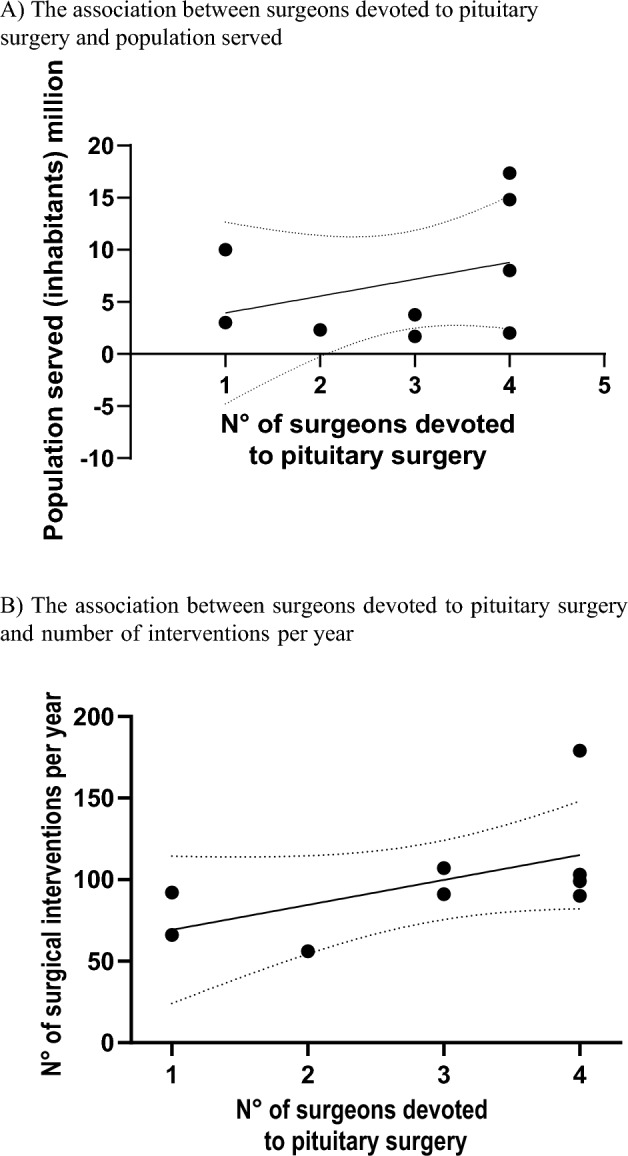
The association between surgeons devoted to pituitary surgery and **A** population served and **B** number of interventions per year as reported by the centers

Overall, a median of 94 (50–200) pituitary interventions per center were performed per year. The median number of transcranial and transsphenoidal (TS) operations per year were 3 (0–16) and 87 (40–200), respectively (Fig. 2). Distribution of surgical interventions per year according to underlying tumor type is provided in Table 2. A linear relationship between number of dedicated surgeons and either population served or number of interventions per year was observed (Fig. 3). In the two centers in which only one active expert neurosurgeon was reported, the workload exceeded 50 and 100 operations per year, respectively.

**Table 2:** Distribution of surgical interventions for pituitary adenomas per center per year according to the underlying tumor type

	Microadenoma (n)	Macroadenoma (n)	Total (n)
Acromegaly	3.6 (0.6–9)	9 (3.3–15.3)	13.3 (6.6–20.6)
Cushing’s disease	5.6 (1.3–17)	2.6 (1–11)	8.6 (2.3–24)
Prolactinoma	2.3 (0–14)	2 (0.3–8)	4.6 (0.3–23)
NFPA	0.16 (0–13.6)	34.5 (16.6–70)	35.3 (24.3–70.3)
TSH-secreting adenoma	0 (0–2)	0 (0–1.6)	0.3 (0–4.6)

Data are presented as median (min–max)

*NFPA* non-functioning pituitary adenoma, *TSH* thyroid stimulating hormone, *n* number

##### Outcomes

- Remission rates

As expected, reported post-surgical remission rates were generally higher for microadenomas than macroadenomas. Interestingly, similar rates of remission were reported for different types of microadenomas, ranging from a median of 74% for prolactinomas to 100% for thyroid-stimulating hormone (TSH)-secreting adenomas. Remission rates for macroadenomas ranged from 49% in acromegaly to 100% in TSH-secreting adenomas. This latter number may be influenced by the low number of patients followed in each center.

- Complications

**Fig. 4:**
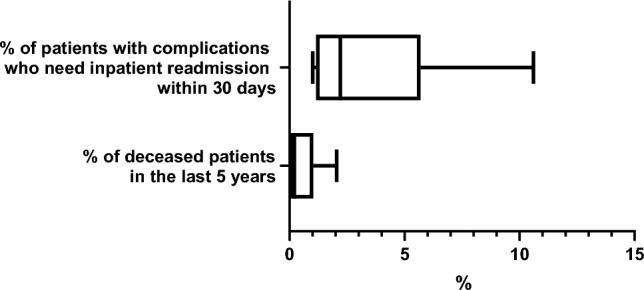
Rate of hospital readmission and mortality after pituitary neurosurgery

Cumulative acute complications occurred at a median rate of 1.45% (1.01–45.7). The rate of inpatient readmission after surgical interventions within 30 days was 2.2% (1–10.6) and mortality rate directly related to surgical intervention in the last 5 years was 0% (0–2.5) (Fig. 4).

**Fig. 5:**
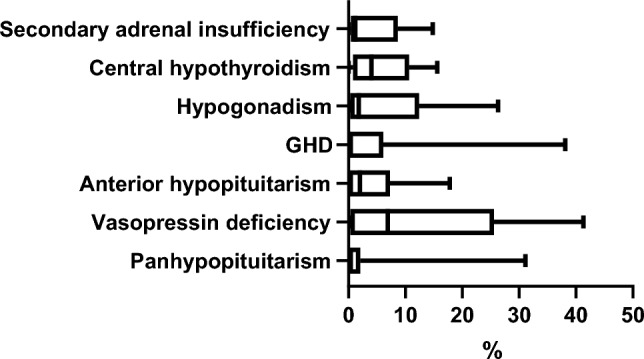
Rates of different types of post-surgical hypopituitarism reported by the centers

Persistent newly onset vasopressin deficiency was reported at a median rate of 6.9% (0–41.3) of patients, growth hormone (GH) deficiency (GHD) in 1.1% (0–38.1), secondary hypogonadism in 3.65% (0–26.3), secondary adrenal insufficiency in 1.6% (0–14.8), central hypothyroidism in 3.65% (0–15.6), anterior hypopituitarism in 2.25% (0–17.8); panhypopituitarism after pituitary interventions was reported in 0.3% (0–31.1) (Fig. 5).

#### Endocrinology

##### Personnel and activity volume

**Fig. 6:**
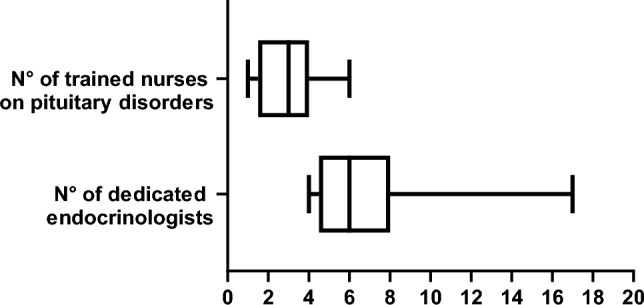
Personnel serving in Endocrine Units

**Fig. 7:**
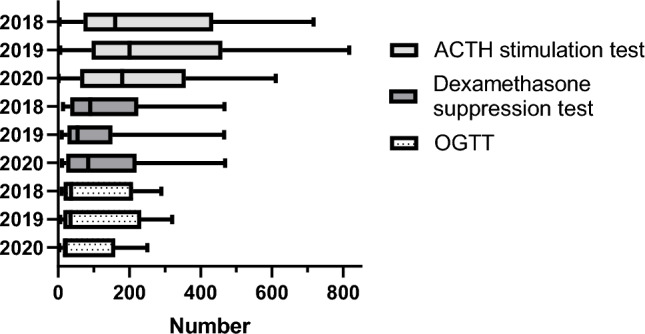
Dynamic Tests subdivided by type and year reported in Endocrine Units

Median number of dedicated pituitary-focused endocrinologists was 6 (4–17) and dedicated pituitary nurses were 3 (1–6) (Fig. 6). Median number of patients with pituitary disorders managed annually in the endocrinology units was 1403 (855–1874). The median ratio between the number of endocrinologists and neurosurgeons per center was 2 (1–6). Overall, 1335 (342–4230) dynamic tests were performed, most frequently the ACTH stimulation test, followed by dexamethasone suppression test and oral glucose tolerance test (OGTT, Fig. 7).

##### Outcomes

Cumulatively, a median of 281 (52–563) patients with hypersecreting pituitary adenomas were reported to be under specific medical treatment, including a similar number of patients with acromegaly, 82 (52–331) and prolactinoma, 97 (25–288). Conversely, only a median of 15 (3–100) patients with medically treated Cushing’s disease were reported. Median cumulative control rate of pituitary patients under medical treatment was 79% (10–100%). Median control rate for Cushing’s disease was 75% (10–100%) with high variability among centers, but only slightly lower than that obtained for acromegaly, which was 83% (64–93%) and much more consistent among centers.

##### Data communications and trials

There was a median of 16 (8–30) publications per year in peer reviewed journals related to pituitary disorders. Median number of invited lectures, abstracts and oral communications per year were 16 (2–19), 12 (0–20) and 3 (0–5), respectively. A median of 4.5 (0–14) ongoing clinical trials on pituitary disorders and a median of 2 (0–4) completed trials per year were reported.

#### Support units

##### Neuroradiology

**Fig. 8:**
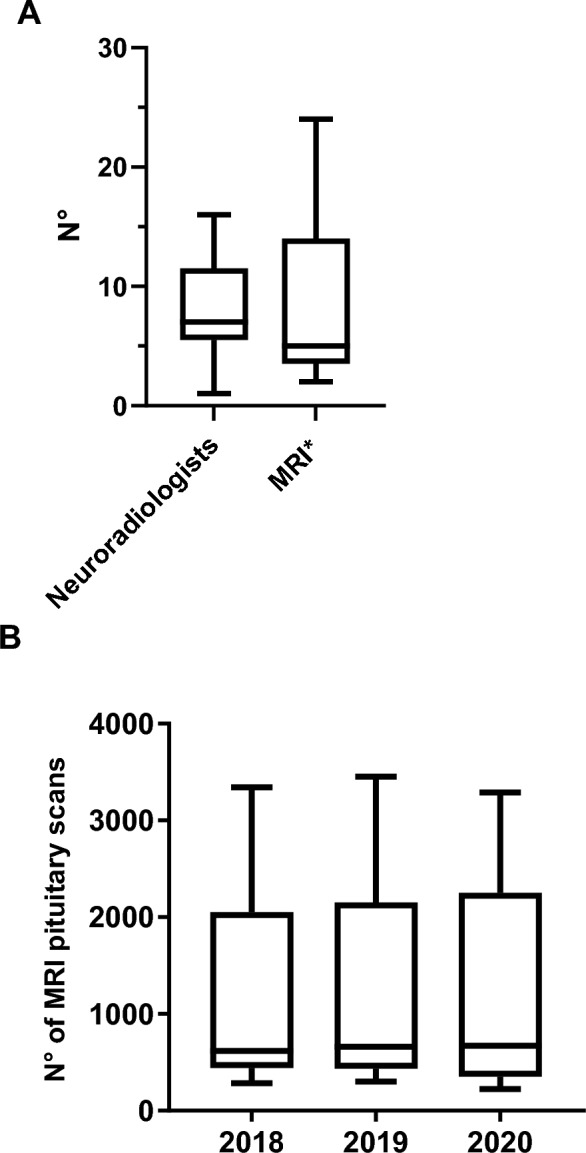
Neuroradiology Unit data (**A**, available personnel and MRI machines * with at least 1.5T field strength and **B**, MRI pituitary scans per year)

Median number of neuroradiologists reported in participating centers was 7 (1–14) with a median number of available magnetic resonance imaging (MRI) machines (1.5 T or above) of 5 (2–24). The median cumulative number of neuroradiological procedures performed was 59,248 (4829–333,081). The median annual number of pituitary scans [MRI and computed tomography (CT)] was 1035 (225–3453) and median annual number of pituitary contrast MRI scans was 810 (125–3411) (Fig. 8). The median annual number of thin-collimation CT scans was 93 (6–2430). A median of 3 (0–20) selective bilateral venous sampling of the inferior petrosal sinus (IPSS) procedures per year was performed.

**Fig. 9:**
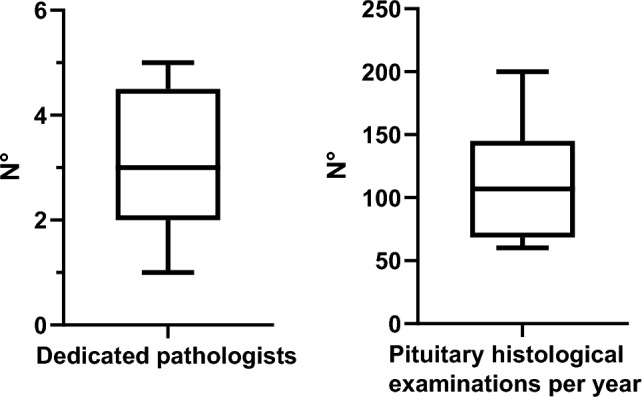
Neuropathology Unit data of personnel and activity

##### Neuropathology

Median number of dedicated pituitary pathologists was 3 (1–5), and 107 (60–200) pituitary histological examinations were performed per year (Fig. 9). All centers performed routine histological assessments including pleomorphism, giant cells, inclusions, inflammatory changes, stroma, hemorrhage, vascular features, mitosis rate and proliferative index (i.e., Ki67) as well as routine pituitary hormone stains, including ACTH, prolactin, GH, TSH, luteinizing hormone (LH), follicle-stimulating hormone (FSH), and additionally in most cases α-subunit (except for two centers). There was heterogeneity regarding availability of modern molecular testing. In fact, one center performed assessment of granulation (sparsely/densely) only upon specific request. Seven centers assessed transcription factors routinely; however, one center performed them only upon specific request and one center did not assess them at all. All centers except one performed somatostatin receptor immunostaining. In all centers tumor specimen banking was available.

##### Radiation therapy

Median reported number of dedicated radiotherapists/radiation-oncologists was 3 (2–5). Number of pituitary stereotactic radiotherapy and radiosurgery interventions per year were 5.3 (2–35) and 4.3 (0–60), respectively; whereas conventional radiotherapy procedures were almost completely abandoned in surveyed centers (median varying from 1 to 5 yearly in last 3 years, with many centers reporting no or 1 procedure per year).

##### Other support units

A median of 2 (1–5) neuro-ophthalmologists, and 3 (1–30) neuro-oncologists was reported by the candidate centers. A median number of 64 (17–272) collaborating non-endocrine specialists (including cardiologists, sleep and bone experts) was reported.

### Proposed validated numerical criteria

Based on the core data analysis, the SEB proposed a synopsis of the main criteria to be used in an accreditation process (Table 3). It included timelines for the accreditation process, as well as activity data for neurosurgery, endocrinology and other support units. The median for each item is referred to as preferred, and the low limit of range as acceptable criterion.

**Table 3:** Synopsis of proposed core criteria to be used for PTCOE accreditation process

Timelines for accreditation process	Preferred	Acceptable
Time for data submission (days)	60	90
Time for re-submission (days)	11	80
Time for referee evaluation (days)	12	65
Time for total evaluation (days)	90	240

Neurosurgery	Preferred	Acceptable
N of dedicated surgeons	3	1
N of pituitary nurses	3	0
Population served	3.7 million	1.5 million
N of pituitary surgical interventions per center	100	50
% of patients with complications who need inpatient readmission within 30 days	< 2%	< 10%
% of deceased patients in the last 5 years	< 0.5%	< 2%

Endocrinology	Preferred	Acceptable
N of endocrinologists	6	4
N of trained nurses	3	1
N of patients	1400	850
N of dynamic tests	1300	350
N of publications in international peer reviewed journals related to pituitary disorders	16	8

Supporting units	Preferred	Acceptable
N of neuroradiologists	7	1
N of dedicated pathologists	3	1
N of radiotherapists/radio-oncologists	3	2
N of neuro-ophthalmologists	2	1

Interestingly, whereas an accreditation process for a putative PTCOE (without considering the time for preparation of the process) can be performed ideally in 3 months, it realistically may take about 8 months. The preferred number of dedicated neurosurgeons is 3, whereas 1 dedicated surgeon does seem to be acceptable. Moreover, although 100 surgical procedures per year per center are preferred, our data suggest that even 50 interventions per year could be acceptable particularly when only one expert neurosurgeon is active in a center (Table 3). Acute post-surgery complications, including mortality and readmission rate, should preferably be negligible or nonexistent, but acceptable rates could be up to 10% of patients with complications requiring readmission within 30 days after surgery (Table 3). Four pituitary endocrinologists are preferred in a PTCOE and the total population of patients with pituitary disorders followed-up in a PTCOE should not be lower than 850 (Table 3). Finally, it appears that at least one dedicated neuroradiology, pathology, and ophthalmology expert dedicated to pituitary diseases and at least two expert radiation oncologists are needed (Table 3).

## Discussion

The PTCOE concept has been developed to meet the worldwide need of objectively identifying centers that optimally support patients harboring pituitary adenomas. This need has increased due to progress in neurosurgical procedures, medical therapies for hormonally-active pituitary adenomas [13], diagnosis and treatment of hypopituitarism [14], and management of systemic complications [15, 16]. PTCOEs should provide patient-centric approaches based on multidisciplinary teams, led by dedicated experts in surgical techniques and medical treatments [9].

This urgent need could generate self-appointed reference centers with a wide heterogeneity in quality of care provided, which might create uncertainty and confusion among patients and health authorities about centers effectively granting the best care for these rare diseases.

Theoretical criteria for defining PTCOEs published by a Panel appointed by the Pituitary Society in 2017 [9] required validation in real-life practice before being accepted globally and used for PTCOE accreditation. To this end, we designed a voluntary pilot study involving several high-volume tertiary pituitary care centers selected by a panel of experts appointed by the Pituitary Society. Initially, participating centers had to pass quality checks by external referees. Data provided by each center were then used to benchmark the activities and infer numerical thresholds for PTCOE definition.

Adequate assessment of each center took longer than expected since it required collection of a substantial volume of information by dedicated personnel to extract and synthesize (during the pandemic) requested data. Moreover, an accreditation process also requires dedicated personnel devoted to a significant organizational effort to evaluate requests for PTCOE accreditation. A further organizational burden may be the need for on-site visits to verify data reliability. Our study did not include a site visit due to COVID-19 restrictions [17].

Criteria for excellence of the endocrine component of the PTCOE [18, 19] were qualitatively defined by a Pituitary Society Panel in 2017. However, limitations included lack of quantitative definitions of the criteria, due to the absence of reference data in the literature [9]. In this regard, our study provides the first systematic attempt, based on large volume of data produced by widely recognized pituitary centers. Interestingly, we were able to define that a PTCOE should have at least 4, preferably 6, dedicated endocrinologists expert in pituitary diseases. In this regard, in any forthcoming accreditation process, specific site environment, such as support staff and percentage effort in clinical care of each endocrinologist should be taken into account. In fact, with progressive decline of in-hospital practicing endocrinologists worldwide, there is a risk that only a few centers could grant this type of effort, unless more resources are allocated and endocrine units dedicated solely or predominantly to pituitary disease are established. Moreover, despite the key role of nurses dedicated to pituitary disease their number is already limited in large centers surveyed and they may not be available in some countries. Based on our data, one such nurse could be acceptable for a PTCOE but, due to global nurse shortages, this criterion may require further consideration.

A typical high-volume pituitary center should encounter > 1000 pituitary patients per year with 850 being the minimal threshold. Since dynamic tests are typically performed in the pituitary center environment [20] and endocrine patients are rarely hospitalized, the number of dynamic tests performed per year appears to be a good criterion to benchmark the volume of activity of the endocrine unit in a PTCOE, which should be > 300 tests/year. This threshold may allow us to assess experience with either pituitary hyperfunctioning adenomas or hypopituitarism.

Endocrinologists are key for achieving remission or control of pituitary diseases [21–26]. Efficacy of medical treatment for secreting pituitary adenomas has been mainly evaluated retrospectively [27, 28]. Therefore, since expertise in optimal use of all available medical tools is required [9], a reasonable criterion for quantifying this parameter is rate of control achieved. Clearly, the same definition of biochemical control should be used by each center, consistently with recent guidelines [15, 16] to allow homogeneous assessment and to avoid variability by us observed in reporting remission in Cushing’s disease. Interestingly, the cumulative degree of biochemical control was quite homogeneous among centers and similar, if not better than that reported in literature [29].

Relevantly to the 2017 criteria [9], a quantitatively good track record of relevant publications in peer-reviewed journals (minimum 8 per year) is required for PTCOEs allowing evaluation and benchmarking of clinical and research activities. In general, outcome studies led by the endocrinologists, also involve surgeons and radiation oncologists. Clearly, specific guidelines for defining publication standards for PTCOEs should be established. PTCOE participation in clinical trials on new molecules offers an additional opportunity for patients not responders to standard therapies and allows to gain experience for future treatment [30], once it becomes generally available. Our results suggest that surveyed centers are involved in a median of at least four clinical trials every year. However, it is interesting to note that one center in our survey was not involved in any trial.

Although recent advances in medical treatment of pituitary tumors have been stellar, undoubtedly a PTCOE is dependent on the presence of a dedicated and excellent neurosurgical group [31, 32] with surgical experience and caseload having significant role in achieving optimal results in addition to tumor-related factors [7, 31, 33, 34]. In a review of 1215 acromegaly UK patients [35], surgical outcome improved after directing care to a small number of subspecialists. Similarly, in a cohort from Japan, increased surgical success for acromegaly from 37 to 81% with transition to single surgeon strategy was reported [36]. Surgical expertise requires specific training at a high-quality center, performing a large number of pituitary interventions each year after training in neurosurgery, and ongoing activity.

Considering that most centers serve a fixed number of inhabitants, the solution proposed in the 2017 statement was to concentrate pituitary neurosurgeons at PTCOEs covering needs of a region [9]. It was also proposed that an ideal reference center could be formed by two to four expert neurosurgeons serving a population of 2.5 to 5 million inhabitants, with a proportional increase in the work load [9, 37], which is confirmed by the linear association we observed between number of neurosurgeons and either population served or number of interventions reported per year. This intrinsically validated the information provided by each Center based on which three neurosurgeons should preferably be devoted to pituitary surgery. The objective ideal population served by a neurosurgery unit in our study is 3.7 million, but greater than 1.5 million is the minimum acceptable, consistent with a previous report [7]. Although the 2017 statement raised the issue that an organization based on a single pituitary surgeon might have drawbacks, our objective results showed that a single surgeon can be considered acceptable. In this regard, PTCOEs may also offer important support to pituitary-surgery experts who work in specialized clinics in neurology and neurosurgery with no availability in house of expert endocrinologists.

The only numerical criterion provided in the 2017 statement concerned the need for at least 50 pituitary procedures every year, data that were based on previous neurosurgical audits and were higher than the 25 annual intervention rates previously proposed. Importantly, results from previous surveys suggest that most neurosurgeons perform pituitary surgery infrequently, and are unable to develop a sufficient clinical experience [38]. Based on our data, 50 procedures per center per year represent an acceptable number in a PTCOE. In fact, high surgical volume translates into more favorable outcomes particularly in invasive adenomas, reoperations for recurrent adenomas, giant adenomas, and microadenomas such as in Cushing’s disease that are not visible on MRI [7].

Efficacy and safety are both important quality markers for surgery [38, 39]. Postsurgical remission rates in pituitary adenomas in our centers were similar to those reported in literature. This may be explained by the fact that only centers with expert surgeons publish in peer-reviewed journals [40, 41]. However, post-surgical remission rates reported for microadenoma in Cushing’s disease were only slightly lower than in acromegaly, whereas in published series, they have been reported to be either higher, similar, or lower [8, 16, 32, 42–45]. Data from surveyed centers may be adversely impacted by the higher percentage of more challenging patients referred from other less specialized centers, where surgical procedures were either not possible or unsuccessful in achieving remission.

In a large US survey on 958 neurosurgical complications of pituitary surgery, these occurred significantly more frequently among respondents with less experience [38]. Based on our results, acute side effects of surgery in a PTCOE (including mortality) should be well below 2%, although post-surgical hypopituitarism may be underdiagnosed, particularly GHD [46]. Readmissions rates after pituitary surgery may vary between 7.2 and 8.5% [47–49]. Thus, < 2% of patients with complications should require inpatient readmission within 30 days. This minimally acceptable threshold is consistent with literature, although a selection bias could exist since most experienced neurosurgeons deal with most challenging cases, who are at higher risk of acute complications [38].

Despite current progress in imaging methods, it can still be challenging to localize a pituitary adenoma, especially in Cushing’s disease [50]. Based on our data, at least one experienced neuroradiologist with work volume of at least 200 pituitary scans a year (and more than 100 contrast MRI per year) is required in PTCOEs. The collaboration with experienced neuroradiologists is also relevant for IPSS, although apparently quite rarely performed [51, 52].

Our results suggest that at least one pituitary-experienced pathologist with a workload of at least 60 pituitary examinations annually is required in PTCOEs. Interestingly, assessment of novel molecular markers is not homogeneously available, even among high volume centers. Therefore, application of novel techniques was not included in synopsis of current criteria for accreditation although, in the future, assessment of molecular markers and transcriptions factors will be likely required in PTCOE.

Computer-assisted techniques performed by expert radiotherapists in treatment of pituitary tumors might offer a fully individualized multimodal therapeutic option [42, 53–56]. However, in surveyed centers, conventional radiotherapy was almost completely abandoned, likely due to negative risk/benefit balance [55, 57]. Moreover, although preferred and minimum numbers of dedicated pituitary radiotherapists according to our data are 3 and 2, respectively, numbers of pituitary stereotactic radiotherapy and radiosurgery procedures per year were reported to be 5 and 4, with minimal thresholds being 2 and 0; respectively. This may reflect the progressive paradigm shift in treatment of pituitary adenomas with increasing preference for more effective and well-tolerated medical therapies. This also raises the question if the requirement of at least two expert radiotherapists in a PTCOE could be reduced to one, given also progressively reduced availability of experts in the field.

In addition to above support units, the contribution of other specialties is necessary to establish a PTCOE [9]. At least one available experienced neuro-ophthalmologist is required, although in some centers computerized visual field examination is carried out by endocrinologists, particularly in non-secreting adenomas [58] or in special conditions (e.g., pregnant women with macroprolactinoma). Moreover, progressively more frequent use of chemotherapy for invasive refractory pituitary adenomas [59] would require the presence of at least one neuro-oncologist in PTCOEs.

This study has several limitations as it relied on self-reported data and did not include on-site visit. Nonetheless, it is unlikely that highly reputable centers, which volunteered for the study, provided inaccurate data. However, among the difficulties we encountered was a misunderstanding of some items in the survey, which needed clarifications from the SEB and revised responses from centers. Therefore, those items would be reformulated for the next steps. The survey asked centers to report the total number of operations performed. Therefore, for the two sites with one neurosurgeon the specific workload was interestingly consistent with the 2017 statement [9], i.e., > 50 procedures per year in both cases. For centers with more than one active expert neurosurgeon we could not precisely define the individual surgical workload. However, we infer that also in these centers, as in the two with one neurosurgeon, the leading operator of the team should fulfill the 2017 criteria, performing the majority of operations with others serving as back-ups or assistants. Nevertheless, during the next phase of the accreditation process, specific information on the activity of each neurosurgeon should be required. The questionnaire did not address for other unexplored areas, including control rate in medically treated prolactinoma, since this aspect was not considered crucial for PTCOE definition, being well managed by general endocrinologist [60], according to guidelines [61]. Moreover, side effects of medical treatment [62] or more detail on services important for PTCOE (such as cardiology, neuropsychology, or bone units) [63] were also not recorded. We believe that a compromise should be reached to obtain core information from centers in a reasonable time frame. Our study demonstrates that the accreditation process may require up to eight months for well-organized centers. This period appears reasonable to report current activity data that may reflect contemporary situations of evaluated centers.

Numerical criteria were statistically extrapolated from collected data using the median and the low limit of range as references for establishing preferred and acceptable thresholds. This statistical extrapolation may be biased by the number of centers evaluated. We also observed wide variability for some activities reported by different Centers. Moreover, only the number of surgical interventions and not other information concerning endocrine, or support units could be validated and normalized for the number of dedicated specialists or population served. Clinical outcomes were collected by experts, and it is likely that in parallel with accreditation of PTCOEs, assessment of other clinics/universities with no PTCOE will be required in future through prospective regional registries and/or through health insurance database analysis. However, our applied methodology allowed us to exclude outlier larger numbers, potentially raising the bar too high for a realistic definition of PTCOE, applicable globally such as European Reference Network on Rare Endocrine Conditions in the EU.

In conclusion, this is the first study to survey and evaluate the activity of several high-volume pituitary centers. This effort internally validated by ad hoc reviewers allowed for transformation of previously formulated theoretical criteria for PTCOE definition to precise numerical criteria based on real-life evidence. Fulfillment of at least the acceptable level of criteria derived from our synopsis would be necessary to achieve accreditation as a PTCOE. As these proposed criteria are accepted by the scientific community, structure for managing accreditation process by the Pituitary Society as an accrediting body would need to be established through a cost-effectiveness analysis also with input from nonmedical experts familiar with developing and implementing accreditation standards. Further steps would optimize the process, such as testing it on a limited number of non-pre-selected centers to assure high standards, objectivity, and transparency. Finally, these steps will lead to the development of identical databases in all centers and allow a rapid, easy, yearly update while forming a robust basis for research and for possible periodic re-evaluation over time of each PTCOE.

### Supplementary Information

Below is the link to the electronic supplementary material.

Supplementary file1 (XLSX 38 kb)

Supplementary file2 (PDF 421 kb)

## Data Availability

The datasets generated during and/or analysed during the current study are not public but are available from the corresponding author on reasonable request.

## Abbreviations

ACTH: Corticotropin
Co-PI: Co-Principal Investigator
COVID-19: Coronavirus disease 2019
CT: Computed tomography
Endo-ERN: European Reference Network on Rare Endocrine Conditions
EU: European Union
FSH: Follicle-stimulating hormone
GH: Growth hormone
GHD: Growth hormone deficiency
IPSS: Inferior petrosal sinus sampling
LH: Luteinizing hormone
LINAC: Linear accelerator
MRI: Magnetic resonance imaging
NFPA: Non-functioning pituitary adenoma
OGTT: Oral glucose tolerance test
PI: Principal Investigator
PTCOE: Pituitary Tumors Center of Excellence
SEB: Scientific Evaluating Board
TS: Transsphenoidal
TSH: Thyroid-stimulating hormone
