# Supplementary material for: Pilot study to define criteria for Pituitary Tumors Centers of Excellence (PTCOE): results of an audit of leading international centers
Source: Pituitary. 2023 Aug 28;26(5):583–96. doi: 10.1007/s11102-023-01345-0 (PMC10539196; doi:10.1007/s11102-023-01345-0)
Supplement: Supplementary file 2 — Supplementary file2 (PDF 421 kb) [file 11102_2023_1345_MOESM2_ESM.pdf]

Evaluation form for PTCOE validation criteria study.

CONFIDENTIAL

We really thank you for accepting to revise data provided by PTCOE candidate.

We kindly ask you to score every item from 1 (poor) to 5 (excellent).

GENERAL-INTRODUCTION

Center facilities

1 = poor	2 = borderline	3 = good	4 = very good	5 = excellent

Clinical multidisciplinary activities

1 = poor	2 = borderline	3 = good	4 = very good	5 = excellent

Education/formation multidisciplinary activities

1 = poor	2 = borderline	3 = good	4 = very good	5 = excellent

ENDOCRINOLOGY UNIT

Clinical activities

1 = poor	2 = borderline	3 = good	4 = very good	5 = excellent

Research/publication/congressual activities

1 = poor	2 = borderline	3 = good	4 = very good	5 = excellent

Trials

1 = poor	2 = borderline	3 = good	4 = very good	5 = excellent

NEUROSURGERY UNIT

Clinical activities

1 = poor	2 = borderline	3 = good	4 = very good	5 = excellent

Complications

1 = poor	2 = borderline	3 = good	4 = very good	5 = excellent

Research/publication/congressual activities

1 = poor	2 = borderline	3 = good	4 = very good	5 = excellent

Trials

1 = poor	2 = borderline	3 = good	4 = very good	5 = excellent

NEURORADIOLOGY UNIT

Facilities

1 = poor	2 = borderline	3 = good	4 = very good	5 = excellent

Clinical activities

1 = poor	2 = borderline	3 = good	4 = very good	5 = excellent

NEUROPATHOLOGY UNIT

Facilities

1 = poor	2 = borderline	3 = good	4 = very good	5 = excellent

RADIOTHERAPY – RADIO ONCOLOGY UNIT

Clinical activities

1 = poor	2 = borderline	3 = good	4 = very good	5 = excellent

OTHER UNITS

Availability of different clinical experts and clinical exams

1 = poor	2 = borderline	3 = good	4 = very good	5 = excellent

General comments

Max. 2000 characters
